# Simvastatin-loaded liposomal nanoparticles as treatment for adenomyosis in a patient-derived xenograft mouse model: a pilot study

**DOI:** 10.1080/01443615.2025.2502083

**Published:** 2025-05-09

**Authors:** Rachel Michel, Kathleen L. Vincent, Gregory W. Kirschen, Massoud Motamedi, Jamal Saada, Jinping Yang, Bulent Ozpolat, Gokhan S. Kilic, Mostafa A. Borahay

**Affiliations:** a Department of Gynecology & Obstetrics, Johns Hopkins University, Baltimore, MD, USA; b Department of Obstetrics and Gynecology, University of Texas Medical Branch, Galveston, TX, USA; c Department of Obstetrics & Gynecology, University of Pennsylvania, Philadelphia, PA, USA; d Department of Ophthalmology and Visual Sciences, University of Texas Medical Branch, Galveston, TX, USA; e Department of Anesthesiology, University of Texas Medical Branch, Galveston, TX, USA; f Department of Nanomedicine, Houston Methodist Research Institute, Houston, TX, USA; g Department of Experimental Therapeutics, The University of Texas MD Anderson Cancer center, Houston, TX, USA

**Keywords:** Adenomyosis, simvastatin, nanoparticles, animal

## Abstract

**Background:**

Adenomyosis is a common gynaecological condition where ectopic endometrial glands and stroma grow within the myometrium. This condition has a high clinical burden impacting those afflicted with debilitating symptoms including heavy painful periods. Simvastatin is an oral hydroxymethylglutaryl-coenzyme A (HMG-CoA) reductase inhibitor, typically used to treat hyperlipidaemia. Simvastatin has recently shown promise for treating gynaecological conditions such as endometriosis and uterine fibroids with nanoliposomal formulations demonstrating improved efficacy. In this pilot study, we tested simvastatin-loaded liposomal nanoparticles on xenografted adenomyosis tissues in a patient-derived mouse model.

**Methods:**

We surgically inserted oestrogen/progesterone pellets into mice, followed by adenomyosis tissue xenografts 15 days later. Mice were then randomised into three groups: control, simvastatin, and simvastatin-loaded liposomal nanoparticles (simvastatin-NP). We quantified the changes in adenomyosis xenograft size weekly using a calliper as well as ultrasound imaging 28 days after treatment, prior to sacrifice. We also measured the proliferation of biomarker Ki67 in the xenografted tissues using immunohistochemistry after animal sacrifice.

**Results:**

Treatment with simvastatin-NP significantly reduced volume and weight of adenomyosis xenografts while attenuating Ki67 expression when compared to the control and simvastatin groups.

**Conclusions:**

This pilot study demonstrates promising improved efficacy of simvastatin delivered via liposomal nanoparticles. However, larger studies are needed to fully explore the potential of simvastatin-NP in adenomyosis.

## PLAIN LANGUAGE SUMMARY

Adenomyosis is a condition where the inner lining of the uterus exists within the muscle wall of the uterus, resulting in painful periods, heavy bleeding, and bloating. Despite adenomyosis’ debilitating symptoms, treatment options remain limited. Previous research suggests simvastatin – a medication used to treat patients with high cholesterol – is a promising treatment for gynaecological conditions. Therefore, in this study, we implanted adenomyosis tissue from humans into mice to test the efficacy of two different forms of simvastatin – regular simvastatin and simvastatin in nanoparticle form – in reducing adenomyosis lesion size. We found that simvastatin in nanoparticle form significantly reduced the adenomyosis lesion volume and weight in mice. We then examined the tissue for a marker of cellular proliferation and found that it was significantly reduced in adenomyosis tissue treated with simvastatin in nanoparticle form. Therefore, our results suggest that simvastatin in its nanoparticle form should be further investigated as a potential treatment option for adenomyosis.

## Introduction

Adenomyosis is a gynaecological condition where ectopic endometrial glands and stroma grow within the underlying myometrium. Typical symptoms of adenomyosis include abnormal uterine bleeding (AUB), painful periods, and pelvic pain (Gunther and Walker 2024). Imaging studies are required for the diagnosis and characterisation of adenomyosis with ultrasound considered the gold standard (Moawad, Fruscalzo, *et al.* 2023). The diagnosis of adenomyosis has historically been limited to post hysterectomy, ex vivo uterine examination, thus the true prevalence and incidence of the condition are unknown (Upson and Missmer 2020). Treatment options for adenomyosis remain limited and often encompass modalities developed for contraception and other benign gynaecological conditions such as uterine fibroids or dysmenorrhoea. These modalities include non-steroidal anti-inflammatory drugs (NSAIDs); progestins (oral, injections, and levonorgestrel intrauterine system); combined oestrogen/progestins oral contraceptives; and hysterectomy which is definitive management but precludes future childbearing (Etrusco *et al.* 2023, Kho *et al.* 2021, Krentel and De Wilde 2022, Moawad, Youssef, *et al.* 2023, Vannuccini and Petraglia 2019). However, these treatments have limited efficacy and are not appropriate for those attempting pregnancy. Thus, there is an urgent need for non-surgical, non-hormonal treatment options.

Simvastatin is a hydroxymethylglutaryl-coenzyme A (HMG-CoA) reductase inhibitor typically used to treat hyperlipidaemia (Talreja *et al.* 2024) with demonstrated efficacy in preventing cardiovascular disease (Chou *et al.* 2022). More recently, scientists have begun to explore its potential in the treatment and prevention of gynaecological conditions, such as endometriosis and uterine fibroids (Afrin *et al.* 2020, 2021, Borahay *et al.* 2014, Bruner-Tran *et al.* 2009, El Sabeh *et al.* 2021). Furthermore, several recent studies have demonstrated improved efficacy of simvastatin-nanoparticles formulations as compared to direct delivery of simvastatin (Borahay *et al.* 2021, El Sabeh *et al.* 2022, Enazy *et al.* 2023, Lulseged *et al.* 2024).

Adenomyosis and endometriosis share several features and often coexist (Vannuccini and Petraglia 2019). Therefore, it’s plausible to infer that because simvastatin has demonstrated efficacy in treatment and prevention of endometriosis, it may produce similar results in adenomyotic tissue (Bruner-Tran *et al.* 2009, Duleba *et al.* 2014, Nasu *et al.* 2009). Thus, our pilot study hypothesised that simvastatin delivered via liposomal nanoparticles – as a method of drug delivery aimed at improving treatment efficacy – would lead to the reduction of adenomyotic lesion size along with lowering of Ki67 as a biomarker of proliferation.

## Methods

### Animal model

Following a protocol approved by the Institutional Animal Care and Use Committee at the University of Texas Medical Branch at Galveston, we purchased 6-week-old female immunodeficient NOG (NOD/Shi-scid/IL-2Rcnull) mice from Taconic (Hudson, NY). These mice are an ideal immunodeficient model as they lack mature T, B, and Natural Killer (NK) cells, allowing them to maintain growth of the human adenomyosis tissue (Borahay *et al.* 2015, 2021, El Sabeh *et al.* 2022). Due to their immunodeficient nature, these mice were housed in isolation within the animal facilities and all experimental procedures were completed under sterile conditions.

### Tissue samples, processing and implantation

We used an analogous model as previously published by our group (Borahay *et al.* 2015, El Sabeh *et al.* 2022), this time with adenomyosis xenografts instead of leiomyoma xenografts. An in-vivo xenograft model was chosen as it can closely mimic human pathophysiology of adenomyosis. Adenomyosis tissue was collected from consented patients undergoing elective hysterectomy at the University of Texas Medical Branch at Galveston. Prior to patient surgery, a screening ultrasound was performed and after surgery, a histopathologic exam was performed to confirm diagnosis of adenomyosis. Samples were collected from the operating room and processed under sterile conditions. A 2-mm Keyes biopsy punch was used to create 2 × 2 × 2-mm cylinders. In order to facilitate tissue growth, each tissue xenograft was submerged in Matrigel^™^ basement membrane matrix obtained from BD Biosciences (San Jose, CA) before insertion into mouse. Two tissue xenografts were surgically implanted subcutaneously in every mouse, one per flank. Incisions were closed with sterile surgical staples. Upon completion of the procedure, mice were monitored for signs of infection and pain. The staples were removed after one week of tissue insertion and the treatment phase commenced.

### Treatment phase preparation

Simvastatin was purchased from Cayman Chemical (Ann Arbour, MI) and activated as previously described (Borahay *et al.* 2015). Activation is necessary to convert the prodrug form to its active form. Simvastatin (25 mg) was first dissolved in 625 μL of ethanol. Next, this solution was added to 935 μL of 0.1 NaOH and heated using a water bath (50 °C) for two hours followed by dilution with water. Using HCl, the pH of the solution was adjusted to 7. Next, the solution was diluted 8-fold using water, reaching a 2 mg/mL concentration. The solution was sterile filtered and stored at 4 °C until ready for use.

Using 1,2-dioleoyl-sn-glycero-3-phosphocholine (DOPC) and 1,2-distearoyl-sn-glycero-3-phosphoryle-thanolamine (DSPE) purchased from Avanti Polar Lipids (Alabaster, AL), we prepared the simvastatin-loaded liposome NPs (simvastatin-NPs). Using a 10:1 mixture and 1:10 mixture, we prepared DOPC/DSPE-Peg2000 and simvastatin-DOPC/DSPE-Peg2000 respectively as previously described (El Sabeh *et al.* 2022). Simvastatin-DOPC/DSPE-Peg2000 was stored lyophilised at 20 °C and dissolved in saline upon use.

### Animal procedures

**Figure 1.**
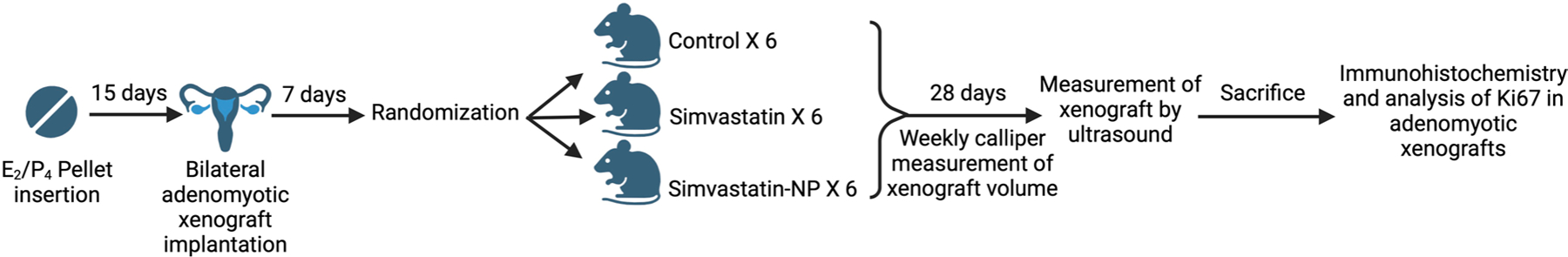
Schematic depiction of the experiment of the experiment. E_2_/P_4_ (oestrogen/progesterone) pellets were inserted into the mice followed by adenomyosis xenografts 15 days after. The treatment phase began 7 days later, mice were randomised to injections of empty vehicle, simvastatin, or simvastatin-loaded liposome nanoparticles (simvastatin-NP). Weekly measurements of xenograft volume were made using a calliper. Mice were sacrificed after 28 days. Immunohistochemistry was then performed on xenografts for Ki67.

Six mice were randomly assigned to each group: control, simvastatin, or simvastatin-NP for a total of 18 mice (Figure 1). Sample size was chosen based on feasibility and our previously published experiments (Borahay *et al.* 2015, El Sabeh *et al.* 2022). Mice were anaesthetised via isoflurane (1–2% by nosecone) for every surgical procedure. We administered postoperative buprenorphine (0.05–0.1 mg/kg) subcutaneously twice daily then as needed for pain control. Sixty-day release pellets with 17ß-estradiol (0.05 mg) + progesterone (50 mg) (Innovative Research of America, Sarasota, FL) were surgically implanted subcutaneously, and then fifteen days later, we implanted adenomyotic xenografts. The pellets were necessary to maintain tissue growth. The control, simvastatin, and simvastatin-NP groups received daily intraperitoneal (IP) injections of 0.2 mL empty vehicle and 0.2 mL of 20 mg/kg/d simvastatin during weekdays (5 days per week), and 0.2 of 20 mg/kg/d simvastatin loaded liposomes IP 3 times weekly, respectively. We based on our administration of simvastatin and simvastatin-NP, and treatment duration on previously published rodent models (Chen *et al.* 2002, Skoglund and Aspenberg 2007, McKay *et al.* 2004, Borahay *et al.* 2015).

The mice were monitored daily after tissue implantation and the xenografts were measured weekly with a calliper for a total of 28 days. Xenograft volumes were also measured once using a high-resolution ultrasound system with a18–38 MHz probe (Vevo 2100; Visualsonics, Toronto, Canada) right before initiating sacrifice. The formula: 0.52 X length X width X height was used to calculate the volume of the xenografts. The animals were sacrificed using isoflurane overdose and cervical dislocation. We then reopened the incision sites and obtained the adenomyotic xenografts. Those initiating injections and xenograft measurements were blinded to the treatment groups. Of note, one mouse died in each of the control and simvastatin groups in the first week and two mice died in the simvastatin-NP groups, at two-week and four-week time points. Their measurements are not included in our analysis.

### Immunohistochemical analysis

The adenomyotic xenografts were placed in 1 mL of a 10% buffered formalin solution for 24 hours and then saved in 70% ethanol kept at 4 °C until staining. Fixed xenografts were used to prepare paraffin sections and immunohistochemistry was performed for Ki67, a proliferation marker of adenomyosis (Matsumoto 1999). ImageJ software was used to quantify the number of Ki67 positively stained nuclei. First, each slide was divided into 4 quadrants. Using a random number generator (1–4), we then drew a 250 × 250 µm region of interest (ROI) in the selected quadrant on each slide. We then performed thresholding on this ROI to only show positively stained nuclei. We then used an automated counting feature in ImageJ to quantify the Ki67 positive nuclei, to reduce potential bias from manual counting. Scientists were blinded to each experimental group when quantifying Ki-67.

### Statistical analysis

We used the robust regression and outlier removal (ROUT) method at Q = 1% to detect and remove outliers from both the adenomyosis xenograft size measurements and the Ki67 soma counts (Motulsky and Brown 2006). One outlier was removed from the xenograft measurements on day 7 in the simvastatin group for a total of *n* = 5 in the control group, *n* = 4 in the simvastatin group, and *n* = 4 in the simvastatin-NP group. Upon removing the outlier, normality was assessed using the Shapiro-Wilk test. Data passed normality test at alpha = 0.05. Data are presented as mean ± standard error of the mean (SEM). The Student’s t-test was utilised to compare adenomyotic xenograft measurements and Ki67 immunohistochemistry results between each treatment to the control and each treatment to each other. P values < 0.05 were considered statistically significant. All statistical analyses were performed in GraphPad Prism and Excel.

### Ethics of experimentation

This study was approved by the University of Texas Medical Branch at Galveston Institutional Review Board (protocol #10–229) and Institutional Animal Care and Use Committee (protocol #1102013).

## Results

### Adenomyotic xenografts growth

**Figure 2.**
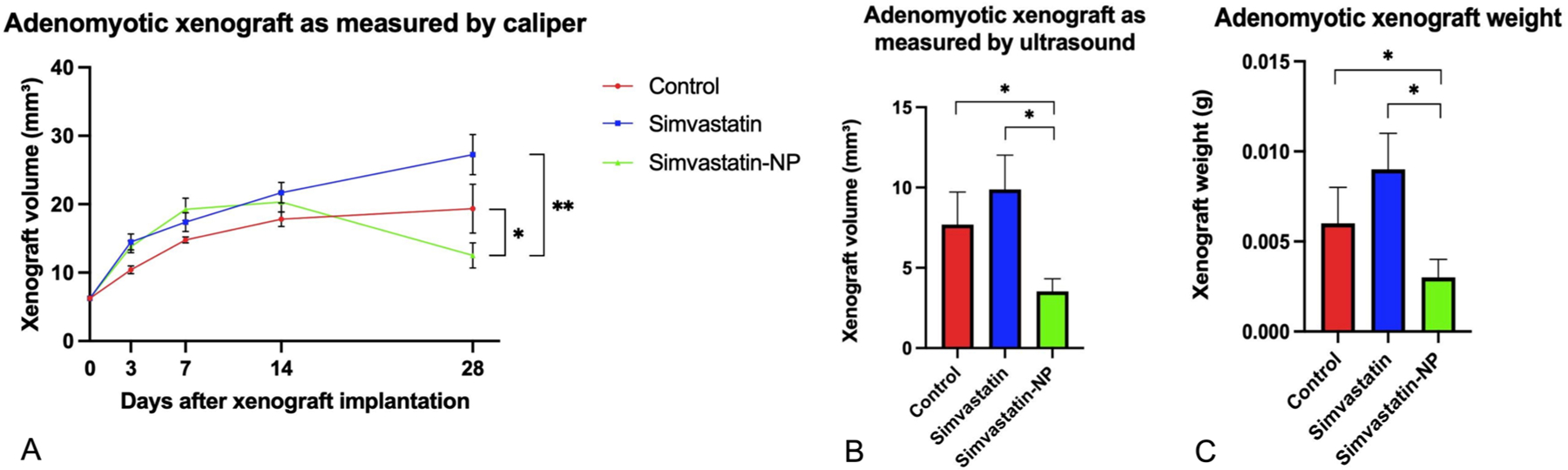
Effect of simvastatin and simvastatin-loaded liposome nanoparticles (simvastatin-NP) treatment on adenomyotic xenograft volume and weight in mouse model. Schematic depiction of the experiment of the experiment. A) Xenograft volume in cubic millimetres as measured weekly by callipers. B) Xenograft volume in cubic millimetres as measured by ultrasound on day 28 prior to sacrifice. C) Xenograft weight in grams taken post sacrifice. Data are presented as mean ± SEM. Asterisk denotes statistical significance at p < 0.05.

We first examined the growth of adenomyosis implants over 28 days in the different treatment groups. As shown in Figure 2A, adenomyotic xenografts grew at similar rates until roughly day 14 when the xenografts from the simvastatin-NP group began shrinking compared to the two other groups. At this same time, the xenografts from the simvastatin group began growing at a faster rate than the control. At the final measurement on day 28 (Figure 2A), the simvastatin-NP group had shrunk to a statistically significantly smaller volume compared to the control group (*p* =0.042) and compared to the simvastatin group (*p* <0.001). On the contrary, the growth of the adenomyosis xenografts in the simvastatin group were not significantly different in this group compared to control (*p* =0.052) (Figure 2A). When measured by ultrasound, the volume of the adenomyotic xenografts demonstrated a statistically significant reduction in size in the simvastatin-NP group compared to the control (*p* =0.044) and compared to the simvastatin group (*p* =0.011), while the volume of the xenografts in the simvastatin group did not show a difference in volume compared to the control (*p* =0.236) (Figure 2B). Lastly, the weight (g) of the xenografts in the simvastatin-NP group was significantly reduced on day 28 compared to control (*p* =0.037) and the simvastatin group (*p* =0.005). The weight (g) of the xenografts in the simvastatin group did not have a statistically significant change compared to control (*p* =0.149) (Figure 2C).

### Intratumoural proliferation

**Figure 3.**
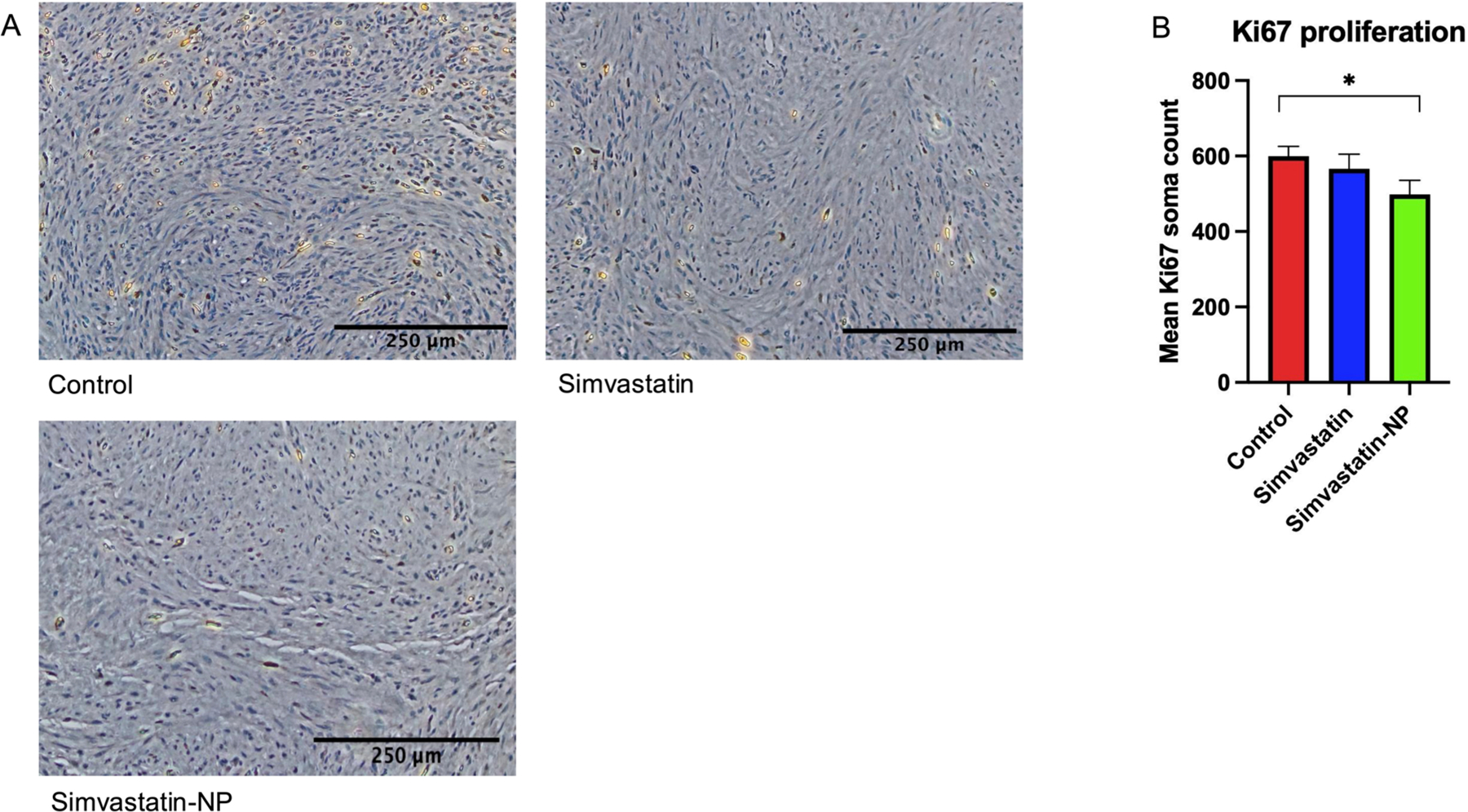
Effect of simvastatin and simvastatin-loaded liposome nanoparticles (simvastatin-NP) treatment on Ki67 proliferation in adenomyosis xenografted mouse model. Adenomyosis tissues collected after sacrifice were placed in 4% formalin solution for 24 hours and then transferred to 70% ethanol until staining. Ki67 expression was quantified using ImageJ. A) Representative slides of Ki67 expression in the control, simvastatin, and simvastatin-NP groups. B) Quantification of Ki67 expression in all three groups. Data are presented as mean ± SEM. Asterisk denotes statistical significance at p < 0.05.

Immunohistochemistry staining was performed on the adenomyotic xenografts to visualise the proliferation marker Ki67. Cells positively stained for Ki67 appear brown in colour while the nuclei of cells negative for Ki67 appears blue. As demonstrated in Figure 3, the simvastatin-NP group significantly reduced the expression of Ki67 compared to the control (*p* = 0.046). The expression of Ki67 did not differ significantly between the simvastatin group and the control group (*p* = 0.473).

## Discussion

Our patient-derived xenograft mouse model demonstrated that 3X weekly repeated IP injections of simvastatin-NPs significantly reduced adenomyotic xenograft volume and weight. Further, treatment with simvastatin-NP for 28 days produced a significant reduction in Ki67, a proliferation marker of adenomyosis. These changes were not present in the simvastatin group, potentially demonstrating the efficacy of simvastatin delivered via nanoparticles resulting in improving simvastatin’s bioavailability.

Drug diffusion into xenografts is driven by multiple factors including the concentration of drug in mouse plasma and the xenograft microenvironment (Saggar *et al.* 2013). Diffusion into adenomyosis tissue likely requires time to reach therapeutic level, thus its effect is delayed from initial administration. This supports why differences in xenograft volume between treatment arms aren’t measurable until day 14. Further, while we discontinued treatment after 28 days – following the timeline of previously published rodent models – we hypothesise that, given the trend we observed in Figure 2, we would continue to see increased adenomyotic shrinkage with continued Simvastatin-NP treatment, although this has yet to be verified (Borahay *et al.* 2015, Neblett *et al.* 2025). In addition, long term experiments of pharmacotherapy in immunocomprimsed mice, especially those who have undergone surgery, is challenging given risk of infection and mortality.

Statins are primarily used to treat hyperlipidaemia through reduction of lipid levels by targeting cholesterol production. Cholesterol is biosynthesized in a multi-step pathway, with the rate limiting step involving the HMG-CoA reductase enzyme catalysing the conversion of HMG-CoA to mevalonic acid, which is then followed by subsequent reactions (Talreja *et al.* 2024). Simvastatin specifically acts on this rate-limiting step, serving as an HMG-CoA reductase inhibitor, resulting in reduced concentrations of cholesterol (Bansal and Cassagnol 2024).

The mechanism of action of simvastatin on the development and growth of adenomyosis in animal models is not yet clear; however, we believe that the results may be explained by the following three mechanisms in which the drug acts including A) inhibition of adenomyotic stromal growth, B) reduced angiogenesis, and C) decreased oxidative stress.

A) Firstly, adenomyosis is a condition characterised by the presence of endometrial glands and stroma within the myometrium (Petraglia *et al.* 2008). Previous work has demonstrated simvastatin’s ability to inhibit endometrial stromal cell growth (Piotrowski *et al.* 2006). Simvastatin may reduce the rate of adenomyotic stromal growth by reducing prenylation of Ras and other GTP-ases involved in cell proliferation and apoptosis, as demonstrated previously in other tissue systems (Bouterfa *et al.* 2000, Danesh *et al.* 2002, Li *et al.* 2002). In addition, tissue from patients with adenomyosis has been shown to have increased expression of matrix metalloproteinase-9 (MMP-9). MMPs are proteolytic enzymes hypothesised to drive stroma remodelling in adenomyotic tissue, causing them to invade surrounding uterine muscle and implant lesions in ectopic zones (Donnez *et al.* 2024). Simvastatin has shown efficacy in inhibiting MMP-3 in cultured human endometrial stromal cells and other cultured cells and tissues (Bruner-Tran *et al.* 2009, Lazzerini 2004, Wilson *et al.* 2005). Since MMP-3 is a known activator of MMP-9, it’s reasonable to suspect that simvastatin may also act as an inhibitor of MMP-9, thus inhibiting growth of endometrial stromal cells and reducing expression of adenomyosis (Johnson *et al.* 2011, Piotrowski *et al.* 2006, Steenport *et al.* 2009, Wilson *et al.* 2005).

B) Next, we propose that simvastatin may reduce angiogenesis in adenomyotic tissue. Simvastatin has been demonstrated to reduce vascular endothelial growth factor (VEGF) secretion – among other angiogenic growth factors – and inhibit angiogenesis in breast and colon cancer cells (Cho *et al.* 2008, Li *et al.* 2017, Wang *et al.* 2018). Further, studies have demonstrated statins’ potential in protecting against endometriotic implants by reducing angiogenesis, as demonstrated by reduced vascularisation of lesions (Bruner-Tran *et al.* 2009, Esfandiari *et al.* 2007). Therefore, due to angiogenic mechanistic similarities between endometriosis and adenomyosis, it’s plausible to deduce that simvastatin may act similarly in adenomyotic tissue by reducing angiogenesis (Donnez *et al.* 2024, Harmsen *et al.* 2019).

C) Lastly, simvastatin’s antioxidant properties may act mechanistically on adenomyotic tissue to reduce oxidative stress. Scientists suspect that exposure of endometrial cells to oxidative stress may contribute to excessive cell growth, creating a conducive environment for establishment of adenomyosis (Kobayashi *et al.* 2020). Statins have demonstrated antioxidant effects in different diseases (Mansouri *et al.* 2022, Verma *et al.* 2022). By modulating the nuclear factor erythroid 2 related factor 2/heme oxygenase-1 (Nrf2/HO-1) pathway – a pathway implicated in the pathogenesis and development of adenomyosis – simvastatin may increase the DNA-binding activity of Nfr2, inducing expression of its target genes and thus allowing protection against oxidative stress (Chen *et al.* 2017, Mansouri *et al.* 2022).

Liposomal nanoparticles are small, biocompatible carriers widely utilised for the delivery of many therapeutics, such as chemotherapeutics agents and immune modulators (Afzal *et al.* 2022). These nanoparticles demonstrate immense potential in the delivery of emerging therapeutics for the treatments of benign gynaecological conditions, with previous studies demonstrating that simvastatin delivered via solid lipid NPs outperformed pure drug powder in an oral rat model, as evidenced by reduced cholesterol values, indicating improved bioavailability (Padhye and Nagarsenker 2013). However, more research is needed to engineer simvastatin-NPs for optimal controlled drug release for the treatment of gynaecological conditions.

To our knowledge, this is the first study that explores the application of simvastatin-NP as a potential treatment of adenomyosis. One study examined the effect of a hypocholesterolemic agent – Probucal – on prevention of development of adenomyosis with promising results in mice. (Zhou *et al.* 2004). Our study lends further evidence that hypocholesterolemic agents may have a role in prevention and treatment of adenomyosis. By utilising an *in vivo*, xenograft animal model, we demonstrate the advantages of slow-release simvastatin via nanoparticles, offering superior therapeutic effects compared to direct simvastatin administration. However, there are several limitations that must be acknowledged. Firstly, the sample size was small, limiting conclusions drawn from this single pilot study. Further, simvastatin and simvastatin-NP concentrations in the adenomyotic xenograft tissue and mouse plasma were not measured, limiting our ability to directly compare absorption rates between the two formulations. Lastly, administering simvastatin only on weekdays, rather than using a sustained regimen, may have decreased the efficacy of naked simvastatin in inhibiting xenograft growth. However, this may better represent ‘typical use’ of the medication rather than ‘ideal use’ and may better represent actual results in real patients, who may skip doses or not take exactly as prescribed.

Also of note, statins may have potential off-target effects on reproductive tissues which may impact uterine and ovarian function. Notably, previous work has showed that statin treatment in animal models resulted in alterations in ovarian morphology, including reduced follicular development and impaired hormone production, which could have long-term implications for reproductive health (Jiao *et al.* 2020). Further research exploring the dose-dependent nature of off-target reproductive effects as well as the potential reversibility of statin-induced reproductive dysfunction are needed to fully elucidate the balance between therapeutic benefits versus potential adverse events when considering simvastatin as a treatment modality.

## Conclusion

Despite limitations, the current study demonstrates the potential advantages of using slow-release nanoparticle to enhance the efficacy of simvastatin for the treatment of adenomyosis, as a promising therapeutic approach.

## Data Availability

The data that support the findings of this study are available from the corresponding author, Mostafa Borahay, upon reasonable request.
